# Yeast-Derived Nucleotides Enhance Fibroblast Migration and Proliferation and Provide Clinical Benefits in Atopic Dermatitis

**DOI:** 10.3390/ijms25052890

**Published:** 2024-03-01

**Authors:** Sergi Segarra, Ivica Bošnjak, Igor Mioč, Bojana Čurčija, Vlatka-Antonija Csik, Srećko Krešić, Jessica Romero-Rueda, Anna Rodríguez, Daniel Martínez-Puig

**Affiliations:** 1 R&D Bioiberica S.A.U., Av. dels Països Catalans, 34, 08950 Esplugues de Llobregat, Spain; dmartinez@bioiberica.com; 2 Veterinarska Ambulanta Bošnjak & Mioč doo, Dinka Šimunovića 2A, 21000 Split, Croatiaigor.mioc@gmail.com (I.M.); 3 Veterinarska Ambulanta Luna doo, Trg Matije Gupca 48, 42000 Varaždin, Croatia; luna.vet.vz@gmail.com; 4 Veterinarska Ambulanta Ljubimac doo, Ivanićeva ul. 19, 10000 Zagreb, Croatia; ljubimac.ambulanta@gmail.com; 5 Veterinarska Ambulanta RiVet doo, Istarska 15, 51000 Rijeka, Croatia; praksa.rivet@gmail.com; 6 Health & Biomedicine Department, LEITAT Technological Center, 08005 Barcelona, Spain; jromero@leitat.org (J.R.-R.);

**Keywords:** nucleotides, RNA, canine atopic dermatitis, yeast extract, *Saccharomyces cerevisiae*, atopic disease

## Abstract

Nucleotides, glycosaminoglycans, and omega-3 essential fatty acids (O3s) could be used for improving skin health, although their modes of action, alone or in combination, are not yet fully understood. To gain some insight into these mechanisms, we performed two in vitro tests and one in vivo pilot trial. The effects on human dermal fibroblast proliferation and migration were evaluated with the following compounds and combinations: 0.156 mg/mL O3s, 0.0017 mg/mL hyaluronic acid (HA), 0.0004 mg/mL dermatan sulfate (DS), 0.0818 mg/mL nucleotides, and [O3s + HA + DS] and [O3s + HA + DS + nucleotides] at the same concentrations. In both in vitro assays, adding nucleotides to [O3s + HA + DS] provided significant improvements. The resulting combination [O3s + HA + DS + nucleotides] was then tested in vivo in dogs with atopic dermatitis by oral administration of a supplement providing a daily amount of 40 mg/kg nucleotides, 0.9 mg/kg HA, 0.18 mg/kg DS, 53.4 mg/kg EPA, and 7.6 mg/kg DHA. After 30 days, the pruritus visual analog scale (pVAS) score was significantly reduced, and no adverse effects were observed. In conclusion, the combination of nucleotides plus glycosaminoglycans and O3s could serve as a useful therapeutic alternative in skin health applications.

## 1. Introduction

Nucleotides are low-molecular-weight compounds that are the building blocks of DNA and RNA. They can be found naturally in all foods of animal and vegetable origin as free nucleotides or nucleic acids and are important for many physiological processes in living organisms [1,2,3]. Under normal conditions, de novo endogenous synthesis serves as the main source of nucleotides in animals, but nucleotide supply becomes conditionally essential in certain situations where demand increases, such as periods of physiological stress, immunosuppression, infection, and certain diseases. In these instances, this pathway is not sufficient. In such cases, and in several animal species, it has been reported that exogenous nucleotide supplementation leads to improved biological functions and has several health benefits, including immunity modulation, infection resistance, promotion of growth and development, maintenance of intestinal and liver functions, and enhanced cell proliferation and differentiation [4,5,6,7].

Products obtained from brewer’s yeast extract are rich in nucleotides. Among other potential applications, they could be used in animals and humans to enhance immune function and for skin health [8,9]. As an example of a particular yeast-derived nucleotide extract used in this way, several previously published investigations support the usefulness of Nucleoforce^®^, a proprietary brand of nucleotide-rich yeast extract developed by Bioiberica S.A.U. (Palafolls, Spain), in modulating immune responses. It is obtained from primary fermented yeast extract, *Saccharomyces cerevisiae*, and besides containing a high concentration of free nucleotides, it is a highly sustainable product as it can be produced through a fermentation process following a circular bioeconomy approach [5].

Atopic dermatitis is a clinical syndrome that affects both people and animals, including dogs. Canine atopic dermatitis (CAD) shares many similarities with atopic dermatitis in people because, in both cases, skin barrier impairment, cutaneous dysbiosis, and immunological dysfunction are key elements involved in its pathogenesis [10,11,12,13,14,15,16,17,18]. CAD requires long-term management, combining topical and systemic therapies, which often results in a highly complex and expensive regime for pet owners [19]. This causes not only discomfort and distress for the dog but also generates stress and a negative impact on the quality of life of the pet’s owner [20]. 

Among the treatment options for CAD, oral supplementation with omega-3 fatty acids (O3s) has been shown to be effective by decreasing inflammation and normalizing the lipid profile in the stratum corneum of the skin [21,22,23,24,25]. Both nucleotides and O3s are considered immunonutrients. The potential to modulate the activity of the immune system by using specific nutrients is termed immunonutrition, an approach closely associated with possible clinical improvements [26,27].

Glycosaminoglycans (GAGs), such as hyaluronic acid (HA) and dermatan sulfate (DS), play a role in skin disease and wound healing processes and can contribute to maintaining skin homeostasis and improve skin physiology [28,29]. There have been reports on the beneficial effects of the particular HA matrix ingredient Dermial^®^ (Bioiberica S.A.U., Palafolls, Spain) used in vitro [30,31,32,33]. Dermial^®^ contains a high concentration of HA (60–75%), DS, and collagen, and reports describe its enhancing action on proliferation and migration of both dermal fibroblasts and epidermal keratinocytes and its stimulation of the production of collagen types I and III, elastin, and GAGs, which, in turn, support the maintenance of the skin’s structure and moisture content. These activities may lead to clinical improvement if used in patients with atopic dermatitis, both human and canine.

Targeting the immune response has been suggested as an approach to developing new therapies for atopic dermatitis [34]. Since nucleotides modulate the immune response and have been shown to improve different parameters involved in the functioning of the immune system, using them in atopic dermatitis-like conditions could provide additional benefits. Also, the proliferation and migration of fibroblasts is an important part of the wound healing process, and these cellular mechanisms play a pivotal role in the progression of dermatological conditions with impaired skin barrier function, such as atopic dermatitis, as well as in skin lesions and tissue repair [35,36,37]. Nucleotides may also contribute to improving said processes and eventually lead to the optimization of the clinical condition of these patients. Additionally, and given the growing concern regarding the rise in antibiotic resistance [38,39], modulating the immune response with nucleotides might seem like a suitable alternative approach to promote a more rational use of antimicrobials, especially if we consider the latest publications arguing that atopic dermatitis is associated with skin dysbiosis rather than with secondary infections [10,11,40,41].

Despite existing scientific evidence pointing towards the usefulness of nucleotides, GAGs, and O3s for skin health, their modes of action, alone or in combination, are not yet fully understood. To gain insight into these mechanisms, we performed two in vitro tests and one in vivo pilot trial. Our research hypothesis was that adding nucleotides to a combination of GAGs and O3s would result in an enhanced action with potential applications in skin health, especially for atopic dermatitis. Based on such a hypothesis, our aim was to evaluate the effects of nucleotides, HA, DS, O3s, and their combinations on cell proliferation and migration in vitro as well as their clinical efficacy and safety in CAD.

## 2. Results

### 2.1. Cell Viability

**Figure 1:**
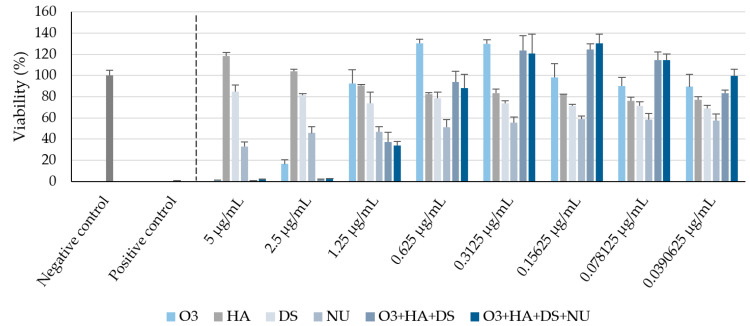
Cell viability percentages (mean ± SD) after 48 h in each experimental condition, compared with non-treated cells, in the cytotoxicity assay. O3s: omega-3 fatty acids, HA: hyaluronic acid, DS: dermatan sulfate, NUs: nucleotides. Negative control: non-treated human dermal fibroblasts (HDFs) maintained in assay medium; positive control: sodium dodecyl sulfate (SDS)-treated HDFs.

The results from the cytotoxicity studies are shown in Figure 1.

These data allowed the selection of the appropriate test concentrations for in vitro wound healing and proliferation studies, which were as follows: 0.156 mg/mL O3s, 0.0017 mg/mL HA, 0.0004 mg/mL DS, and 0.0818 mg/mL nucleotides (NUs). Moreover, a combination of O3s + HA + DS (weight ratio 65:0.73:0.15) and a combination of O3s + HA + DS + NUs (weight ratio 65:0.73:0.15:34.09) were also tested. The final concentrations and ratios between the test compounds were based on the cell viability data, but the dosages of the active ingredients to be potentially used in vivo were also considered so that such combinations would result in a product for oral administration which could be feasible and affordable.

### 2.2. Fibroblast Migration

**Figure 2:**
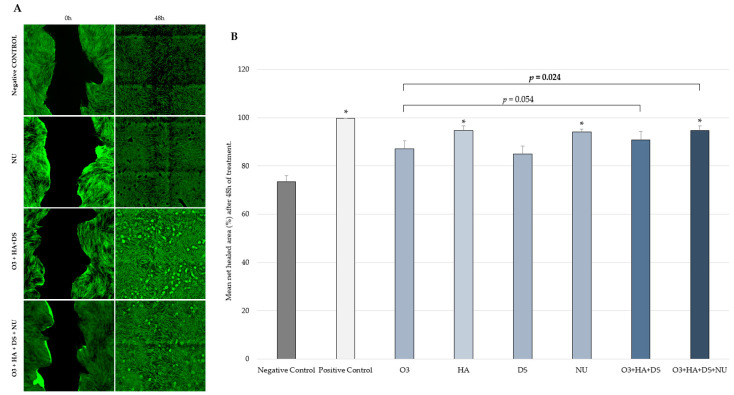
(**A**) Digital microscopy images taken during the in vitro wound healing study (scratch test) on HDFs using an Olympus TIRF-ScanR automated inverted microscope (Leica) and a 4× objective lens (40× total magnification), just after scratching the HDF monolayer (0 h) and after 48 h of treatment. (**B**) Net healed area (mean ± SD) on HDF culture 48 h after scratching and treatment. Percentage vs. T0h of each experimental condition evaluated in triplicate. * *p* < 0.05 vs. negative control. Negative control: non-treated HDFs maintained in assay medium; positive control: HDFs treated with TGF-β1.

After 48 h, a significantly increased (*p* < 0.05) mean human dermal fibroblasts (HDF) migration rate compared to the negative control was seen with HA (mean ± SD = 94.66% ± 1.94), NUs (mean ± SD = 94.07% ± 1.19), the combination [O3s + HA + DS + NUs] (mean ± SD = 94.65% ± 1.98), and the positive control (mean ± SD = 99.84% ± 0.02), but not with the combination [O3s + HA + DS] (mean ± SD = 90.88% ± 3.49). Compared to O3s alone, a significantly higher mean HDF migration rate was achieved with the combination [O3s + HA + DS + NUs] (*p* = 0.024) but not with the combination [O3s + HA + DS] (*p* = 0.054) (Figure 2). No other significant differences were observed.

### 2.3. Fibroblast Proliferation

**Figure 3:**
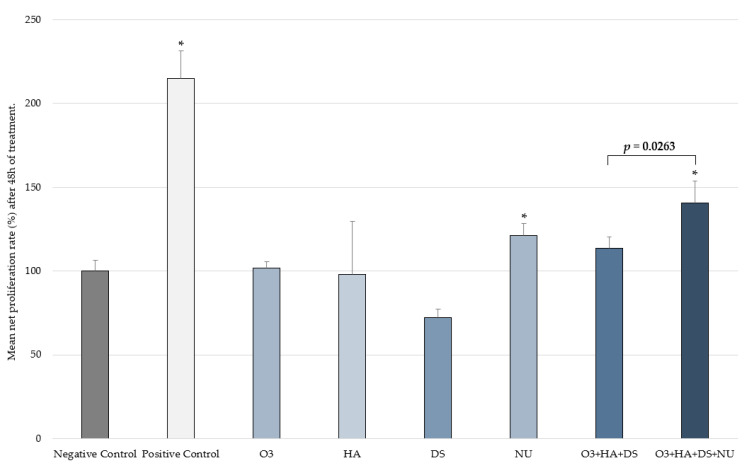
Proliferation rate (mean ± SD) in the control groups and the two combinations after 48 h. Percentage vs. T0h of each experimental condition evaluated in triplicate. * *p* < 0.050 vs. negative control. Negative control: non-treated HDFs maintained in assay medium; positive control: HDFs treated with TGF-β1.

After 48 h, a significantly increased (*p* < 0.05) mean HDF proliferation rate compared to the negative control was seen with NUs (mean ± SD = 121.34% ± 7.04), the combination [O3s + HA + DS + NUs] (mean ± SD = 140.7% ± 13.19), and the positive control (mean ± SD = 214.72% ± 16.61). The combination [O3s + HA + DS] showed a non-significant increase (mean ± SD = 113.4% ± 6.86; *p* > 0.05). When the effects of the two different combinations of compounds were compared, HDF proliferation enhancement by the combination [O3s + HA + DS + NUs] was found to be significantly better than that of the combination [O3s + HA + DS], achieving a significantly higher percentage of HDF proliferation (*p* = 0.0263) (Figure 3). No other significant differences were observed.

### 2.4. Clinical Efficacy in Atopic Dogs

A total of eight dogs were included in the study. None of them were lost during the follow-up, and they all completed the study for 30 days. This total included five males and three females (37.23 ± 11.08 months of age) of different canine breeds: mixed breed (*n* = 3), French bulldog (2), Labrador retriever (*n* = 1), Doberman (*n* = 1) and Boxer (*n* = 1). 

**Figure 4:**
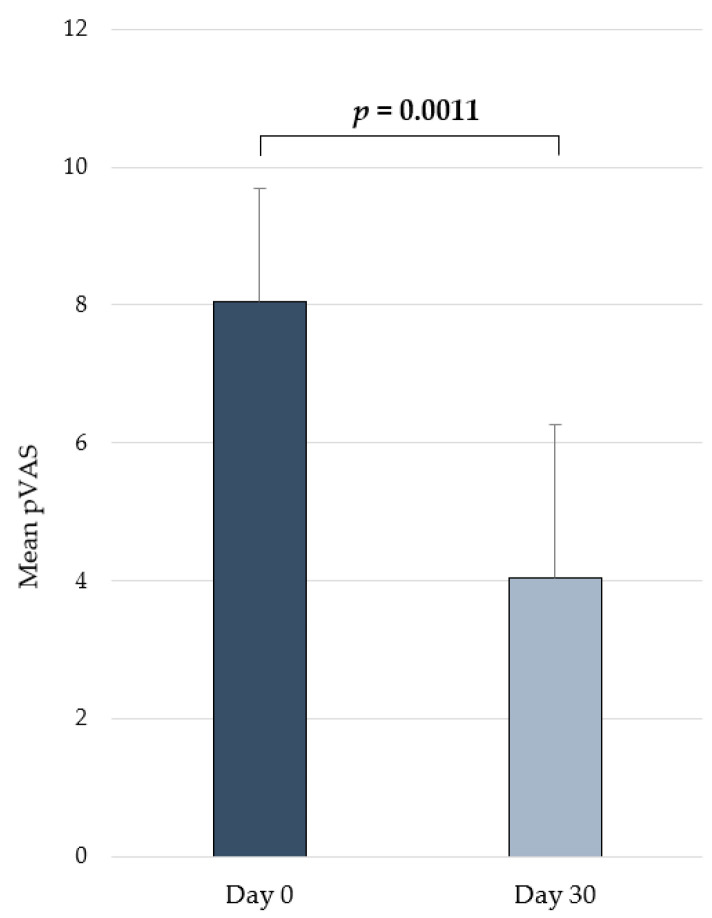
Changes in pVAS (mean ± SD) over time in dogs from the in vivo pilot study receiving the daily oral administration of the supplement containing the combination [O3s + HA + DS + NUs].

A significant reduction in the pVAS (*p* = 0.0011) was seen between 0 (mean ± SD = 8.05 ± 1.65) and 30 (mean ± SD = 4.04 ± 2.22) days (Figure 4). The product was well tolerated, and no adverse effects were observed during the course of the study.

## 3. Discussion

Atopic dermatitis is still a complex, incurable disease affecting a large population of dogs, leading to a marked reduction in quality of life for both dogs and their owners, causing emotional and financial strain [19,42]. Nucleotides could potentially serve many different clinical applications. More specifically, yeast-derived nucleotide-rich extracts could be useful in the food and feed industries, as well as in biotechnology, cosmetics, and medical industries, including indications for pruritus, skin inflammation, and wound healing [8]. Within the veterinary medicine field, dietary nucleotides also have many potential applications. In the particular case of CAD, novel therapies should focus on topical delivery, improving the skin barrier function and enhancing treatment compliance by the owners [20,34,43,44,45,46]. Therefore, solutions could arise from alternatives that help maintain the patient in remission or extend the length of the proactive phase of the disease. 

Findings from the in vitro studies described herein show that nucleotides can significantly enhance both the migration and proliferation of HDFs. Although HA alone also significantly improved cell migration, its effects on proliferation did not reach the level of statistical significance at the tested concentrations. HDF migration and proliferation were not significantly increased either when the other compounds were tested on their own. Interestingly, after the impact of combining these compounds was evaluated, an enhancing effect was discovered for the addition of nucleotides to a combination of GAGs and O3s, as it resulted in a significant increase in both the proliferation and migration rate of HDFs. Consequently, a combination of nucleotides, GAGs, and O3s could be useful for the treatment or prevention of pathologies that affect dermal health in small animals and would also be indicated as a complement to therapies focused on restoring skin integrity by improving wound repair. It should be mentioned that unlike what occurred in the proliferation test, in the scratch assay, the net healed area was greater with the combination [O3s + HA + DS + NUs] than with [O3s + HA + DS], but this difference did not reach a level of statistical significance. However, when compared to the negative control and O3s alone, despite [O3s + HA + DS + NUs] achieving a significantly greater healed area, this effect was not observed with [O3s + HA + DS].

Fibroblasts and keratinocytes, the main skin cell types, are known to be important elements in the pathogenesis of atopic dermatitis. Their migration and proliferation are involved in the process of re-epithelialization, which is key in skin pathologies where the skin barrier function is altered and in patients suffering from lesions caused either by the pathology itself or by scratching due to itching [35,47]. Hence, the significant effects observed in the in vitro studies described herein are of importance, indicating that the addition of nucleotides significantly promotes HDF proliferation and migration.

From a clinical perspective, pruritus is the main clinical sign of CAD, and although there are many antipruritic therapies available for managing it [48], they have some limitations. For this reason, the significant improvement achieved with the tested combination on the pVAS in our pilot in vivo trial in atopic dogs becomes a relevant finding. In previous publications, immune dysregulation featuring a Th2-polarized response has been proposed as a cause for the development and perpetuation of CAD by promoting humoral immunity. Moreover, in the skin and blood of atopic dogs, alterations in Th1 and Treg immune responses have also been described [10,37,49]. Nucleotides, and specifically the Nucleoforce^®^ nucleotide-rich yeast extract, have been shown to modulate the immune response in a way that enhances a Th1 cell immune response in diseases with a strong immune base, like leishmaniosis [5,50,51]. It would therefore be reasonable to speculate that a similar modulation might be achieved in patients with atopic dermatitis and, with that, eventually also potentially contribute to the fight against antimicrobial resistance following a One Health approach by sparing the use of antibiotics [39]. What is more, provided that the dog is considered a good animal model for human atopic dermatitis and that this condition affects animals in a similar fashion to that of humans, and also considering that the compounds from this composition can be used in humans, it might make sense to consider this intervention also in people. In such a case, several studies should be performed in order to assess the efficacy and safety of this combination of nucleotides with GAGs and O3s.

It should also be mentioned that, in a recent publication, a study evaluating the effects of the oral administration of nucleotides alone to dogs with atopic dermatitis showed some improvements in pruritus and erythema. It should be noted though that, in that case, the nucleotides were of a different type and were administered on the day of enrollment and then again 14 days later [52], as opposed to the daily administration followed in the study reported herein. Nevertheless, despite the differences between these studies, they both point towards the potential benefits of nucleotides in CAD.

In the particular case of the study presented herein, it should be emphasized that the tested intervention contains dietary nucleotides and O3s. Both nucleotides and O3s are immunonutrients that interact with and modulate the gut microbiota composition and variability [27]. This may have an impact on the systemic inflammatory response and eventually result in clinical benefits for patients suffering from skin conditions.

The main limitation of our in vivo trial was that it was an uncontrolled pilot study, making it necessary to further confirm these benefits in a randomized controlled study using a larger sample size. Another potential limitation would be the lack of in vitro testing of the [O3s + HA + DS + NUs] combination using an inflammatory stimulus which better resembles what occurs clinically in atopic patients. This could have been performed prior to advancing into in vivo testing but might also be performed now as a further investigation in order to better characterize the mechanism of action and the benefits provided by this intervention. And even another path to be explored would be to assess the cytotoxicity of nucleotides in different types of cancer cell lines. Also, based on our findings, the use of this combination of nucleotides, O3s, and GAGs seems to be effective and safe in dogs but could be explored in cats too. Those suffering from feline atopic skin syndrome [53,54] could benefit from a reduction in pruritus as well as from enhanced cell migration and proliferation in the skin. Lastly, and considering the components of this combination and the beneficial effects described in this article, patients suffering from skin conditions other than atopic dermatitis, which also involve an abnormal immune response, skin inflammation, wound healing issues, and altered keratinization processes, could perhaps benefit from it too. In such cases though, further studies would be warranted in order to confirm its efficacy and safety.

## 4. Materials and Methods

### 4.1. In Vitro Studies

Cell proliferation and migration studies were performed on HDFs in vitro, in order to evaluate the effect of several compounds: O3s (Incromega E7010-LG-(LK), Croda Ibérica, S.A., Barcelona, Spain), HA (Bioiberica S.A.U., Barcelona, Spain), DS (Bioiberica S.A.U., Barcelona, Spain), and nucleotides (NUs; Nucleoforce^®^, Bioiberica S.A.U., Barcelona, Spain). HDFs were obtained from human foreskin samples, surpluses from surgery of young donors (0–3 years old), and established by using the standard method of explant growth and enzymatic dissociation of proliferating cells. Cells were propagated and grown in growth medium (GM: Dulbecco’s 1 g/L glucose medium (DMEM; Sigma-Aldrich, St. Louis, MO, USA) supplemented with 10% fetal bovine serum (FBS, PAA Laboratories, Westborough, MA, USA), 2 mM L-glutamine (Lonza, Basel, Switzerland), and antibiotics (100 μg/mL penicillin and 100 U/mL of streptomycin, Lonza, Basel, Switzerland)). For routine subcultivation and propagation of the primary culture, cells were washed twice with phosphate-buffered saline (PBS, pH 7.4, Sigma-Aldrich), harvested with trypsin–EDTA (GibcoTM, ThermoFisher Scientific, Waltham, MA, USA), and counted in a Neubauer chamber before being seeded in a new cell culture flask (Falcon 75 cm^2^, ThermoFisher Scientific, Waltham, MA, USA).

Prior to the proliferation and migration studies, compound cytotoxicity in HDFs was evaluated after cells were treated with increasing doses of compounds and combinations. The results defined the maximum non-cytotoxic concentration of the products in the experimental model. Briefly, cells were seeded in 96-well culture plates at a density of 5500 cells/well and maintained in GM until good cell adhesion was achieved (6–7 h after seeding). Then, cells were maintained overnight in deprivation medium (DM: Dulbecco’s 1 g/L glucose medium supplemented with 0.1% FBS (PAA), 2 mM L-glutamine (Lonza), and antibiotics (100 μg/mL penicillin and 100 U/mL of streptomycin (Lonza))). Thereafter, cells were exposed to 8 different concentrations (5, 2.5, 1.25, 0.625, 0.3125, 0.15625, 0.07812, and 0.03906 mg/mL) of the tested compounds prepared in assay medium (AM: Dulbecco’s 1 g/L glucose medium supplemented with 1% FBS, 2 mM L-glutamine, and antibiotics (100 μg/mL penicillin and 100 U/mL of streptomycin)) for 48 h. At the end of the exposure period, the test solutions were removed, and the cultures were washed with Hanks’ Balanced Salt Solution (HBSS, Sigma-Aldrich) in order to completely eliminate the remaining product. Subsequently, cell viability was assessed using the resazurin Alamar Blue^®^ (Invitrogen, Thermo Fisher Scientific, Waltham, MA, USA) test. Alamar Blue^®^ was applied to the cell culture at 10% (*v*/*v*) in AM. After 2 h of incubation (37 °C, 5% CO_2_), fluorescence was determined using a multi-detection microplate reader (SAFIRE2, TECAN, Männedorf, Switzerland) at 540 nm excitation wavelength and 590 nm emission wavelength. Data were properly recorded and processed to ascertain mean viability (%) and standard deviation (SD) for each experimental condition. Non-treated cells and sodium dodecyl sulfate (SDS)-treated cells were used as negative and positive controls, respectively. Each experimental condition was evaluated in triplicate. The cell viability results allowed the selection of the appropriate test concentrations for the in vitro wound healing and proliferation studies.

An in vitro wound healing assay was performed by testing the abovementioned compounds and combinations in triplicate. Non-treated cells and transforming growth factor beta-1 (TGF-β1)-treated cells (25 ng/mL, PeproTech EC, London, UK) were also used as negative and positive controls, respectively. Primary HDFs were seeded on 24-well plates (Falcon, area of 1.99 cm^2^) at a density of 7 × 10^4^ cells/well in 1 mL of GM. Cells were maintained in growth conditions (37 °C, 5% CO_2_, in saturated humidity conditions) until confluency (3 days). Before scratching, cells were starved for 48 h in DM to synchronize the culture cell cycle. Then, a straight wound of ~2.0 mm width was made across the surface of the confluent cell monolayer using a sterile plastic tip (p1000). The various compounds and combinations were applied immediately to the wounded cultures. HDFs were allowed to migrate back into the wound site in the presence of test products and controls for 48 h. Cultures were visualized immediately after scratching using an Olympus TIRF-ScanR automated inverted microscope (Leica; Wetzlar, Germany) and a 4× objective lens (40× total magnification). Scratch wound margins were visualized, and 30 fields along the wound length were identified. Digital images of the 30 fields were joined to form a full image of the well and recorded for subsequent analysis in order to obtain baseline reference area values (total wound area, prior to treatment (T0h)). After 48 h of treatment, new digital images were recorded from the same fields identified before treatment (T0h), so the same wound field was analyzed in each treatment period assessed. To determine wound healing efficiency, the area of the wound not covered by cells (wound area, μm^2^) was measured at each assessed time point, at T0h and after 48 h of treatment (T48h), using image analysis with Image J software (version 1.50; 26 March 2016). In order to determine the net healed area, the value of the wound area 48 h after scratching was subtracted from the value of the initial wound area for each analyzed field. Then, the mean healed area and standard deviation were calculated for each experimental condition evaluated. The mean percentage of healed area (μm^2^) was determined after 48 h. 

The effect of the products and combinations on cell proliferation was evaluated in an in vitro assay by quantifying bromodeoxyuridine (BrdU) incorporation during DNA synthesis in the replicative phase of the HDFs. Cells were seeded in 96-well culture plates at a density of 5500 cells/well and maintained in GM until good cell adhesion was achieved (6–7 h after seeding). Then, cells were maintained overnight in DM. After deprivation, test compounds and combinations were prepared in AM and incubated with the cells for 48 h. After the exposure period, cell proliferation was evaluated by the Cell Proliferation BrdU ELISA kit (Roche, Basel, Switzerland). After 6 h of incubation (37 °C, 5% CO_2_, saturated humidity conditions), the labeling medium and test solutions were removed, and the cultures were washed with HBSS in order to completely eliminate any remaining product and non-incorporated BrdU. Cells were then fixed, and the DNA was denatured in one step by adding the FixDenat ready-to-use solution provided in the kit. Anti-BrdU-POD conjugate was applied immediately, and after the incubation period, the incorporated BrdU was detected by adding a chemiluminescence ELISA substrate solution for color development and subsequent photometric detection. The reaction product was finally quantified by measuring the absorbance at the respective wavelength of 450 nm. Non-treated cells and TGF-β1-treated cells (25 ng/mL) were used as negative and positive controls, respectively. Each experimental condition was evaluated in triplicate.

### 4.2. In Vivo Testing

An uncontrolled pilot study involving client-owned dogs with naturally occurring CAD was conducted to evaluate whether these compounds could also be effective in vivo and provide skin health benefits. Dogs of any age, sex, and breed were assessed for eligibility if they presented with a history and clinical signs compatible with non-seasonal CAD after other causes of pruritic dermatitis had been ruled out. The diagnosis was made following the criteria of Favrot et al. [55], and prior to entering the study, all eligible candidates also had to undergo an elimination diet for a minimum of eight weeks. Dogs were excluded if they had received O3s, glucocorticoids, oclacitinib, lokivetmab, cyclosporine, or GAGs 8 weeks prior to the study onset. During the course of the study, dogs were excluded if they showed evident signs of intolerance or adverse effects in response to the treatment applied, or a severe worsening of clinical signs. 

Dogs included in this in vivo study received an oral supplement (Atopivet^®^ capsules, Bioiberica S.A.U., Barcelona, Spain) which provides a daily amount of 40 mg/kg NUs, 0.9 mg/kg HA, 0.18 mg/kg DS, and 76.3 mg/kg O3s (53.4 mg/kg EPA and 7.6 mg/kg DHA) for 30 days. The responsible veterinarians performed assessments using the pruritus visual analog scale (pVAS) before (day 0) and after the treatment (day 30). An analysis of variance (ANOVA) test was used for the comparisons between groups at different time points. *p* ≤ 0.05 was considered statistically significant. The results were analyzed using Statgraphics centurion 18, v 18.1.13 (Statgraphics Technologies, Inc., The Plains, VA, USA).

## 5. Conclusions

The results from the in vitro and in vivo tests performed describe the beneficial effects of nucleotides with O3s and GAGs on skin health. As reported herein, the addition of nucleotides to a combination of O3s, HA, and DS significantly enhances HDF proliferation and migration in vitro. Moreover, the resulting composition combining nucleotides with GAGs and O3s provides in vivo clinical benefits in dogs with atopic dermatitis, hence becoming a therapeutic alternative with interesting skin health applications. Further investigations with appropriate randomized controlled clinical trials are needed. This research aimed at exploring the potential positive impact of this new intervention on companion animals, and the use of this intervention for people suffering from dermatopathies may show benefits in quality of life.

## Data Availability

The datasets used and/or analyzed during the studies reported herein are available from the corresponding author on reasonable request.
